# Quality of life in patients submitted to surgical treatment of idiopathic scoliosis

**DOI:** 10.1590/1413-785220152306115026

**Published:** 2015

**Authors:** João Bernardo Sancio Rocha Rodrigues, Nathália Ambrozim Santos Saleme, José Lucas Batista, Igor Machado Cardoso, Charbel Jacob

**Affiliations:** 1 Santa Casa de Misericórdia de Vitória, Escola Superior de Ciências, (EMESCAM), Vitória, ES, Brazil; 2 Hospital Santa Casa de Misericórdia de Vitória (HSCMV), Spine Group, Vitória, ES, Brazil

**Keywords:** Scoliosis/surgery, Treatment outcome, Quality of life

## Abstract

**OBJECTIVE:**

: To evaluate quality of life, using the SF-36, in patients with adolescent idiopathic scoliosis (AIS) who un-derwent surgery for deformity correction, comparing the results in the pre-and post-operative period.

**METHODS:**

: We evaluated 29 patients, 24 female, mean age 14.5 years, all patients had measurement of Cobb angle greater than 50º, and responded to the SF-36 questionnaire preope-ratively and on average two years after surgery.

**RESULTS:**

: There was improvement in all eight domains studied by the SF-36 after surgical treatment, with statistically significant improvement of the domains functional capacity physical aspects, pain and general state. Vitality and mental heal-th were those with the lowest percentage of improvement postoperatively.

**CONCLUSION:**

: Surgical treatment of defor-mity in all AIS improved the functional aspects assessed by the SF-36, representing, in practice, better quality of life for these patients. **Evidence Level II, Prospective Study.**

## INTRODUCTION

Idiopathic scoliosis is the lateral deviation in the frontal pla-ne of the spine larger than 10 degrees, for which there is no established cause, which affects approximately 2-3% of the general population, with a higher prevalence in female adoles-cents. The most common complaint is aesthetical, however, pain, paresthesia, and changes or loss of sphincter balance may also occur.^1^ ^2^

Although the etiology of idiopathic scoliosis remains unknown, there are several multifactorial theories as neuromuscular or con-nective tissue disorders, hereditary factors, changes in sagittal configuration of the spine, asymmetric growth of the limbs and trunk, besides environmental factors, such as nourishment.^3^ ^6^ Studies have shown that untreated scoliosis result in a higher incidence of pain and increasing disability, which can lead to issues at work and marital relations, besides causing respira-tory distress and early death.^7^ ^9^ For more serious cases, it is the orthopedist decision to indicate surgery, aiming to prevent the progression of the disease, correct the curve and maintain the spine balance.^10^ However, even with appropriate treatment established, it is known that in severe deformities there is a sig-nificant negative impact on the patient's quality of life, affecting daily activities common to their age and psychosocial develo-pment of adolescents.^11^

The term quality of life has been used in health disciplines since 1970. It is a multidimensional construct that captures the impact of health status, including disease and treatment in the physical, psychological and social function domains. Usually, the quality of life in health is evaluated through questionnaires because they show greater reliability in treatment evaluation, allowing revealing positive or even negative interference in patients' lives. When analyzing the quality of life of patients with adolescent idiopathic scoliosis (AIS) in the pre- and pos-toperative periods through the SF-36 questionnaire, we believe providing important data on how this disease interferes with the lives of these patients and, with this understanding, to facilitate care and the doctor-patient relationship, increasing patients' adherence to treatment.^12^ ^13^

## MATERIALS AND METHODS

This is a prospective study that evaluated 29 patients with a mean age of 14.53 years old, 24 females, who underwent surgery at the Spine Surgery Group of *Hospital Santa Casa de Misericordia de Vitoria* (HSCMV), Vitória, ES, Brazil. This research project was approved by the institutional Ethics Committee for Research with human subjects of *Escola Superior de Ciências da Santa Casa de Misericórdia de Vitória* (EMESCAM) under number 018/2012. All patients signed the Free and Informed Consent Form (FICF). 

 We used as inclusion criteria all patients with AIS treated at HSCMV with curves over 50°, who responded to SF-36 ques-tionnaire pre- and postoperatively. Exclusion criteria were other causes of scoliosis, patients with curves that received conserva-tive treatment indication, or those who did not have preoperative quality of life evaluation protocols.

Patients were submitted to SF-36 questionnaire preoperatively and on average 24 months after surgery. The SF-36 question-naire to assess quality of life can be self-administered by com-puter, telephone or by a trained interviewer, which contains 36 items that measure mental and physical health components through eight domains: functional capacity, limitations due to physical aspects, pain, general health status, vitality, social aspects, emotional aspects and mental health.

Regarding statistical analysis we initially applied the Kolmo-gorov-Smirnov test, used to assess whether data followed a normal distribution, as shown in Table 1.

The variables functional capacity, general health status, vitality and mental health showed to be normally distributed and cor-related using the Student *t* -test for paired data. For non-normal variables, we used the Wilcoxon test, which is a non-parametric technique equivalent to the Student *t* -test for paired data.^14^

Values of *p* ≤0.05 were considered statistically significant. The statistical analysis was performed using Microsoft Office Excel 2010 software and SPSS (Statistical Package for Social Scien-ces) version 8.0.

**Table 1:**
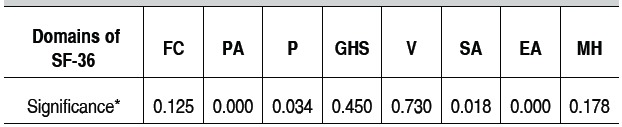
Result of statistical significance (p) of each domain of SF-36 according to Kolmogorov Smirnov test for verification of the data distribution pattern.

*p≤0.05: significant test, non-normal data. FC: Functional capacity; PA: Physical aspects; P: Pain;

GHS: General health status; V: Vitality; SA: Social aspects; EA: Emotional aspects; MH: Mental health.

## RESULTS

**Figure 1:**
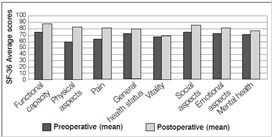
. Comparison between mean values of SF-36 domains on pre and postoperative periods.

The results showed improvement in all eight domains evaluated by the SF-36 questionnaire comparing pre- and post-operative pa-tients undergoing surgery for spinal scoliosis correction. (Figure 1) In Figure 1 we observe a marked improvement in SF-36 average score after surgery for physical aspects functional domain, with an average increase over 20 points. In contrast, the variable vi-tality had the lowest percentage of improvement postoperatively compared to the average before surgery.

By correlating the data obtained from the statistical analysis, we find that among the evaluated domains, functional capa-city, physical aspects, pain and general health status showed statistically significant improvement between periods. (Table 2) Among the eight domains evaluated, functional capacity, physi-cal aspects and pain showed greater level of significance when compared, reflecting an improvement in the practice of daily activities, including vigorous ones, with reduced or no pain or limitations secondary to pain.

Although there is no statistical significance for the domain related to social aspects (p=0.055), the level of significance found very was close to 0.05. Regarding the pain domain, we noticed that 21 patients, equivalent to 72.42% of the sample had some degree of improvement postoperatively, whereas in the vitality domain, only 11 patients noticed improvement after surgery (37.93%).

## DISCUSSION

In this article we used the SF-36 questionnaire to evaluate the quality of life of patients with AIS due to convenience. This questionnaire can be applied to more than 130 conditions, in-cluding spinal-related problems, which may considerably affect the quality of life related to health.^15^ ^18^

When analyzing the general result of surgical treatment of AIS by applying the SF-36 questionnaire, we observed a significant improvement in quality even after two years of surgical correc-tion. We believe that this period after surgery can provide an idea on ​how the surgery can interfere with the quality of life of these patients. Pellegrino and Avanzi, 19 in a similar study con-ducted recently, observed a worsening of pain and functional capacity of patients in the early postoperative period (up to three months), with significant improvement when they were re-evaluated after 12 months of treatment.

**Table 2:**
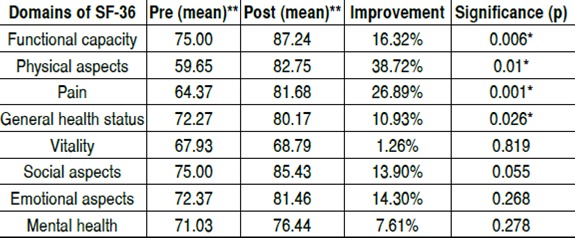
Comparison between the overall averages for each functional domain of SF-36 in the pre- and postoperative period, regarding their respective percentage of improvement after surgery

Pre: preoperative period; Post: postoperative period. *p≤0.05 ** Values may vary on a scale from 1-100, where 100 is the best possible score

An important outcome of this study was a statistically significant improvement in functional capacity, pain and physical aspect, a result similar to that found by Cabral et al.^17^ The literature reports that the incidence of pain in scoliosis is comparable to the incidence in the general population.^1^ In our study, we found improvement in spinal pain in 72.42% of the sample, which leads us to believe that we need to consider its prevalence in these patients. The improvement in physical appearance makes it clear that scoliosis is a physical problem that slightly interferes on vitality and mental health. We, as well, found some improvement in such domains, although not statistically significant. The topic quality of life has become so important in the analysis of postoperative results of IAS that less aggressive surgical tech-niques for treatment have been advocated, such as selective ar-throdesis, where the goal is to perform arthrodesis in as minimum levels as possible, since the spine stiffness in the segment is a constant concern regarding the quality of life of these patients. Despite all the discussion on the topic, we did not yet found in the literature any work that directly shows improvement of quality of life with fewer arthrodesis. What currently exists, and was found in our study, is that surgical treatment of spinal scoliosis, when necessary, improves the quality of life of patients regardless the number of levels on which in which arthrodesis was performed.^20^

## CONCLUSION

Surgical treatment of IAS improved all functional aspects as-sessed by SF-36 questionnaire, representing, in practice, im-provement in the quality of life of these patients.
